# Bradykinesia induced by pallidal neurostimulation in dystonia: clinical risk factors and anatomical mapping

**DOI:** 10.1038/s41531-025-01177-8

**Published:** 2025-10-23

**Authors:** Florian Lange, Diego L. Guarin, Stella Mosert, Berenike Karrasch, Jonas Roothans, Benedikt Weigl, Pavel Navratil, Christine Daniels, Thorsten Odorfer, Gregor Brandt, Philipp Mahlknecht, Joachim K. Krauss, Joachim Runge, Andrea A. Kühn, Günther Deuschl, Jens Volkmann, Robert Peach, Martin M. Reich

**Affiliations:** 1 Department of Neurology, University of Würzburg, Würzburg, Germany; 2 Movement Estimation and Analysis Laboratory, Department of Applied Physiology and Kinesiology, University of Florida, Gainesville, FL USA; 3 Movement Disorders and Neuromodulation Unit, Department of Neurology, Charite-Universitätsmedizin Berlin, Berlin, Germany; 4 Department of Neurology, Faculty of Medicine and University Hospital Cologne, Cologne, Germany; 5 Department of Neurology, Innsbruck Medical University, Innsbruck, Austria; 6 Department of Neurosurgery, Hannover Medical School, Hannover, Germany; 7 Department of Neurology, University Kiel, Kiel, Germany; 8 Department of Brain Sciences, Imperial College London, London, UK

**Keywords:** Medical research, Neurology, Neuroscience

## Abstract

Pallidal deep brain stimulation (DBS) for dystonia can induce bradykinesia. We analyzed retrospective (*n* = 55) and prospective (*n* = 11) cohorts to identify risks and anatomical substrates for this side effect. Bradykinesia was prevalent (60–72%), with female sex, older dystonia onset, shorter disease duration, and crucially, stimulation pulse width identified as key predictive factors. Probabilistic mapping isolated a posterolateral globus pallidus internus “sour spot” for bradykinesia, which was spatially distinct from therapeutic “sweet spots” and demonstrated patient-level predictive power in cross-validation (R² = 0.16, *p* = 0.0013). Kinematic analysis showed reducing stimulation selectively improved movement frequency without altering amplitude. The effect appears mediated by local grey-matter modulation, not major white matter tracts. These findings suggest programming strategies using shorter pulse widths while avoiding the identified sour spot can mitigate bradykinesia without sacrificing antidystonic benefit.

## Introduction

Deep brain stimulation (DBS) of the internal globus pallidus (GPi) is an established and effective treatment for dystonia, a *hyperkinetic* movement disorder characterised by involuntary, patterned muscle contractions, significantly reducing symptom severity and improving quality of life in the majority of patients^1–3^. Clinical outcomes, however, remain variable, and a growing body of evidence shows that therapeutic gains can be accompanied by PD-like *hypokinetic* impairments. Early observations by Ostrem et al. indicated the substantial improvements in dystonia following GPi-DBS were often at the expense of impairments in dexterity and general mobility^4^. Subsequent studies expanded this finding, reporting additional side effects such as micrographia^5^, gait hypokinesia^6^, freezing of gait^7^, and upper limb bradykinesia^8^. Although typically mild, these symptoms can become clinically meaningful, mitigate some of the symptomatic benefits of GPi-DBS, and impact patient well-being^9^. In rare cases, pallidal stimulation has triggered a full Parkinsonian syndrome; Reese et al. described a dystonia patient whose DBS-induced freezing and postural instability met Hoehn and Yahr stage III criteria^10^.

Why should a treatment for a hyperkinetic disorder reproduce hypokinetic features? One possibility is that high-frequency GPi neurostimulation disrupts basal ganglia circuit activity enough to shift neuronal activity towards a Parkinsonian state. This hypothesis is supported by evidence of overstimulation induced beta oscillations, a biomarker of the off-stimulation state in PD patients implanted with GPi-DB, during chronic sensing in the GPi^11,12^. Yet alternative explanations for these PD-like symptoms have also been proposed, including inadvertent subthreshold tetanic stimulation of the adjacency white matter pathways such as the internal capsule^4,13,14^. Clarifying whether these PD-like manifestations represent a distinct pathophysiological entity or merely a spectrum of stimulation-related motor side effects is clinically important. A mechanistic understanding will allow more precise target selection, programming strategies that maximise benefit while minimising bradykinesia, and ultimately a better quality of life for patients treated with GPi-DBS for dystonia.

To address the origin of PD-like symptoms in dystonia treated with GPi-DBS, we performed two complementary analyses. First, a retrospective multicentre cohort study identified demographic and clinical predictors of stimulation-induced bradykinesia. Second, a prospective investigation in eleven cervical dystonia patients used detailed hand-movement kinematics recorded on- and off-stimulation to characterise bradykinesia with millisecond precision. Together, these analyses aim to refine our understanding of DBS-induced PD-like symptoms, improve patient selection criteria, and refine clinical strategies and optimal symptom control in pallidal DBS for dystonia.

## Results

### Description of the retrospective cohort (Cohort I)

We retrospectively analysed data from 86 patients who underwent GPi-DBS implantation for dystonia across multiple European centers. Following the exclusion of 10 patients due to unavailable video recordings and further 21 patients with incomplete neurological examinations for PD motor signs, the final retrospective analysis (Cohort I) included 55 patients (28 males, 27 females). Patient demographics, dystonia classifications, and DBS stimulation parameters are summarized in the Supplementary Table 1.

Among these patients, dystonia was classified as cervical in 23 (41.8%), generalized in 25 (45.5%), and segmental in 7 (12.7%) patients. The mean age of dystonia symptom onset was 37 ± 19 years and patients had lived with dystonia for a mean duration of 15 ± 11 years prior to DBS surgery. The mean age at the time of implantation was 52 ± 14 years. Stimulation parameters varied, with a mean amplitude of 3.4 ± 1.1 mA (range: 1.1–6.0 mA), mean pulse width of 112 ± 43 µs, and mean stimulation frequency of 153 ± 24 Hz.

Postoperative blinded video assessments revealed that 33 out of 55 patients (60%) showed an increase in their Normalized Bradykinesia Score (NBS). This was quantified using the Bradykinesia Difference Score (BDS), where any positive value was counted as worsening. While this high prevalence suggests that subtle parkinsonian-like signs are a common side effect, the clinical impact appears limited. The median change (BDS = 0.250) was small, and the interquartile range (–0.063 to 0.500) indicates that the effect was minimal or even reversed in a substantial portion of the cohort. These findings suggest that while our metric was sensitive to detect minor motor slowing, it does not directly correlate with clinically meaningful disability.

### Description of the prospective cohort (Cohort II)

A detailed prospective clinical and kinematic evaluation was performed in a subgroup of 11 cervical and segmental dystonia patients (2 males, 9 females). Before their participation in this study, these patients had received implantations of various DBS stimulators and electrodes at specialized neurology centers in Würzburg, Cologne, Düsseldorf, and Basel. All participants completed the full set of experimental procedures, with the baseline clinical characteristics and stimulation settings shown in Table 1.

**Table 1:** Demographic, clinical, and deep brain stimulation (DBS) parameters for the prospective patient cohort (Cohort II)

Pat No/Sex	Dystonia Type	Age at Onset [yrs]	Age at Surgery [yrs]	Disease Duration [yrs]	Bradykinesia Items Full Stim.	Bradykinesia Items Half Stim.	TSUI Score Full Stim.	TSUI Score Half Stim.	Mean Amplitude [mA]	Mean Pulse Width [µs]	Mean Frequency [Hz]
P01 / f	CER	36	45	10	9.5	7	4	3	6.8	90	179
P02 / m	CER	33	61	28	8.5	5.5	10.5	8,5	4.7	90	179
P03 / f	CER	18	45	27	6	7	5.5	4	2.45	60	180
P04 / f	CER	50	60	10	13	9	3	5	3.8	40	179
P05 / m	SEG	44	46	2	14.5	10.5	0.5	1.5	3.8	120	170
P06 / f	SEG	50	52	2	5.5	6.5	2.5	6.5	2.65	90	180
P07 / f	CER	4	63	59	8	8	2.5	2.5	4	90	130
P08 / f	CER	61	65	4	18	17	4	6.5	5.25	90	180
P09 / f	CER	55	56	11	20.5	20	3	3	3.4	60	180
P10 / f	CER	39	64	25	10.5	10	4.5	4.5	4	60	179
P11 / f	CER	50	64	9	14.5	9.5	2.5	3.4	4.1	120	130
**Mean Cohort II**		**40**	**56**	**17**	**11.7**	**10**	**3.8**	**4.4**	**4.1**	**82.7**	**169.6**
**Mean Cohort I**		**37**	**52**	**12**	**NA**	**NA**	**NA**	**NA**	**3.4**	**105**	**150**

Values for ‘Bradykinesia Items’ represent the sum of items 3.4–3.8 from the MDS-UPDRS III, assessed under full and half stimulation conditions. The bottom two rows provide mean values for the prospective (Cohort II) and retrospective (Cohort I) cohorts for direct comparison.

*CER* cervical dystonia, *Full Stim.* chronic full stimulation settings, *Half Stim.* stimulation amplitude reduced by 50% from chronic settings, *MDS-UPDRS III* Movement Disorder Society Unified Parkinson’s Disease Rating Scale Part III, *SEG* segmental dystonia, *TSUI* Tsui Dystonia Rating Scale, *yrs* years.

The mean age of dystonia symptom onset was 40 ± 17 years, with a mean age at implantation of 57 years ± 9, and a mean disease duration prior to DBS of 17 ± 17 years. The DBS settings for this cohort were characterized by a mean stimulation amplitude of 4.1 ± 1.2 mA, mean pulse width of 83 ± 25.3 µs, and a mean frequency of 169 ± 19.8 Hz. Additionally, the mean total electrical energy delivered (TEED) was calculated to be approximately 245.2 ± 239.2 µJ.

Clinically, Cohort II exhibited relatively mild dystonia severity under chronic DBS stimulation (mean TSUI score: 3.9 ± 2.6), which was only slightly elevated after acute stimulation amplitude (mean TSUI score: 4.5 ± 2.0, difference not significant). Notably, reducing DBS amplitude acutely led to a small but statistically significant improvement in Parkinsonian bradykinesia-related items of the MDS-UPDRS III (from 11.7 ± 4.9 at full stimulation to 10.0 ± 4.5 at half stimulation, *p* = 0.026). Despite this improvement, these bradykinesia scores remained considerably higher than typical scores reported for healthy, age-matched individuals (see “Discussion”).

Assessments of functional disability and quality of life indicated mild-to-moderate impairment (TWSTRS Disability: mean 7.5 ± 5.4) and a moderate pain severity (TWSTRS Pain: mean 6.3 ± 5.3) before surgery. The cohort also reported a moderate quality of life on the EQ-5D-3L scores (56.4 ± 14.2%). Minimal gait disturbances or freezing were documented (mean FOGQ score: 1.5 ± 1.4 out of a potential 24), indicating negligible gait impairment or freezing of gait in daily life.

### Key factors influencing stimulation-induced Bradykinesia in the retrospective cohort

**Fig. 1:**
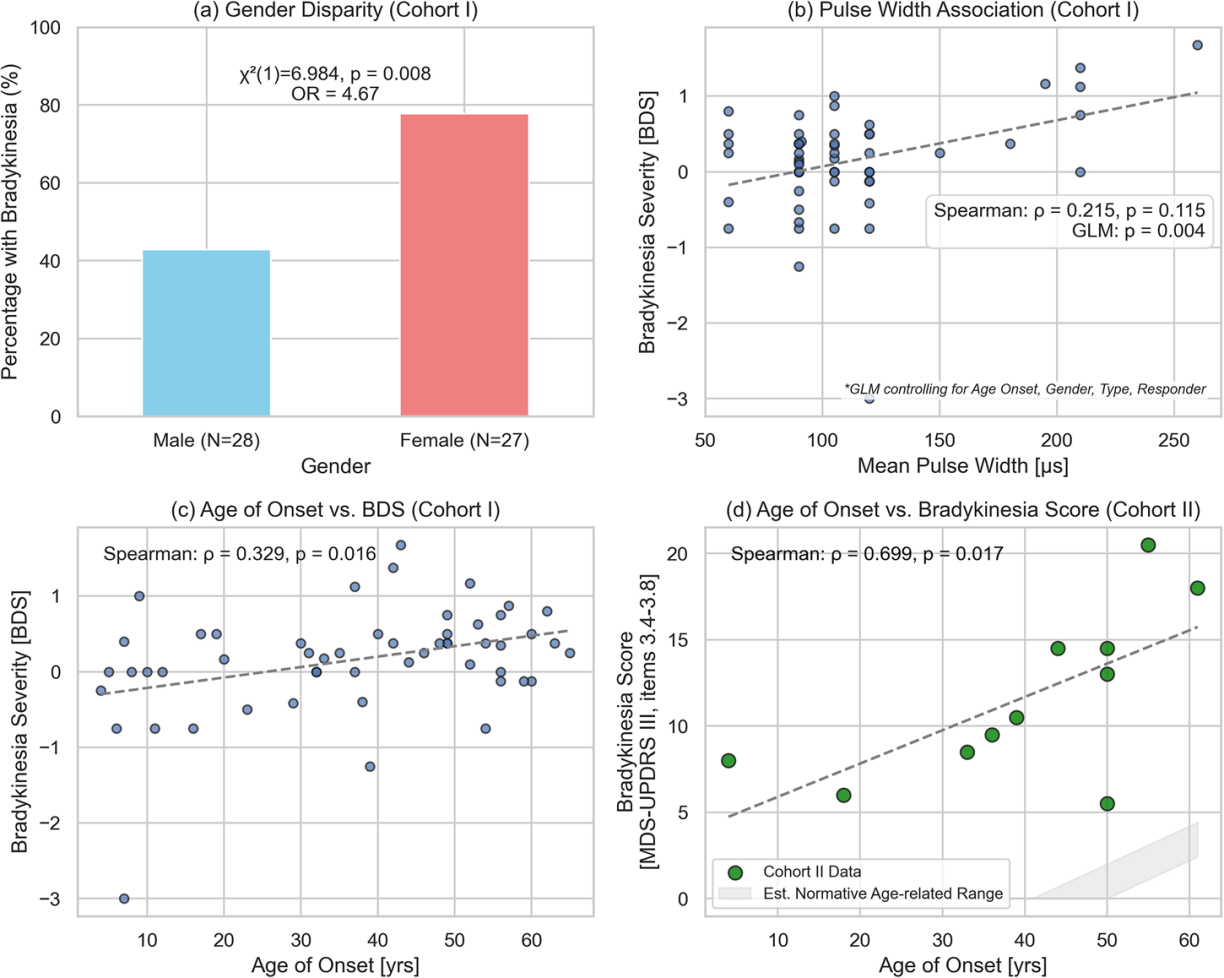
Key factors influencing stimulation-induced bradykinesia in dystonia patients undergoing DBS. **a** Sex Imbalance: A significantly higher percentage of female (77.8%) versus male (42.9%) patients exhibited bradykinesia post-surgery (χ² = 6.984, *p* = 0.008, Odds Ratio: 4.67). **b** Pulse Width Impact (Cohort I): Higher stimulation pulse width significantly predicted increased bradykinesia severity (BDS) in a multivariate GLM (*p* = 0.004). **c** Age of Onset (Cohort I): Older age at dystonia onset was significantly correlated with greater bradykinesia severity (BDS) (Spearman: ρ = 0.329, *p* = 0.016). **d** Age of Onset (Cohort II): A similar significant positive correlation was observed between older age of symptom onset and bradykinesia scores (MDS-UPDRS III) (Spearman: ρ = 0.699, *p* = 0.017). The gray shaded region represents an estimated normative age-related range. This upward trend uses a slope (approx. 0.22 points/year) derived from age-related changes reported in healthy individuals^73^. It is applied here for illustrative contextual comparison to demonstrate how strong the bradykinesia severity in this cohort was compared to any age-related changes.

To identify clinical predictors of stimulation-induced bradykinesia following GPi-DBS, we performed statistical analyses on our retrospective cohort, beginning with univariate tests of association. A significant sex-related difference emerged, with female dystonia patients significantly more likely to develop stimulation-induced PD-like symptoms (χ² = 6.984, *p* < 0.008, Fig. 1a). Specifically, a positive BDS as a surrogate parameter for bradykinetic signs was observed in 77.8% (21/27) of female patients compared to 42.9% (12/28) of male patients, resulting in an odds ratio of 4.66 (95% CI: 1.53–14.22). To investigate whether this disparity could be explained by differences in stimulation programming, we performed a comparison of key stimulation parameters between sexes. A series of Mann–Whitney U tests revealed no significant differences for any parameter: mean amplitude (males: 3.6 mA, females: 3.3 mA; *p* = 0.573), mean pulse width (males: 116.4 µs, females: 107.8 µs; *p* = 0.684), mean frequency (males: 154.7 Hz, females: 152.7 Hz; *p* = 0.819), or the composite Total Electrical Energy Delivered (TEED) (*p* = 0.477).

Next, we investigated the association between disease-related variables and bradykinesia severity using Spearman’s correlation analyses. A higher age at dystonia onset correlated positively with increased bradykinesia severity scores (rho = 0.329, *p* = 0.016; Fig. 1c), Conversely, disease duration prior to surgery demonstrated a significant negative correlation with bradykinesia severity (rho = –0.379, *p* = 0.005), suggesting that patients with shorter disease duration were more susceptible to this stimulation-induced side-effect. It is important to note, however, that while the use of the BDS difference score in this cohort helps mitigate confounding from baseline age-related slowness present before surgery, chronological age at assessment (often linked to onset age and duration) could still influence the susceptibility to stimulation-induced motor changes (see “Discussion”).

Subsequent multivariate modeling aimed to clarify independent predictors of stimulation-induced bradykinesia. Initial analyses revealed significant multicollinearity between several predictor variables, which can lead to unstable model estimates. Specifically, we observed a strong correlation between age at symptom onset and disease duration, and between Total Electrical Energy Delivered (TEED) and mean stimulation amplitude. To construct a robust model, we addressed this by excluding the redundant variables. Disease Duration was excluded due to its inherent mathematical relationship with age of onset. Similarly, as stimulation amplitude is the more direct programmable parameter and the primary determinant of TEED, it was retained in the model while TEED was excluded. The final Generalized Linear Model (GLM) therefore included Age of Onset, Mean Pulse Width, Sex, Type of Dystonia, and Responder status as predictors.

The final GLM analysis yielded an adjusted R-squared value of 0.215, indicating that approximately 22% of the variance in the BDS score could be explained by the included predictor variables. This analysis identified Mean Pulse Width as the sole significant independent predictor of stimulation-induced bradykinesia severity (*p* = 0.004). Notably, Age of Onset, despite its significant univariate correlation with BDS, was not found to be an independent predictor in this multivariate model (*p* = 0.219). Similarly, other variables including Amplitude, Frequency, Sex, Type of Dystonia, and Responder status were not significantly associated with bradykinesia severity in this final model.

### Prospective cohort analysis: influences on stimulation-induced Bradykinesia and its effects on quality of life and disability

To validate the retrospective findings, we prospectively analysed a group of 11 patients. Consistent with our retrospective cohort, we found a robust, and in fact stronger, positive correlation between age of symptom onset and bradykinesia severity (rho = 0.699, *p* = 0.017; Fig. 1d). Similarly, a negative correlation between shorter disease duration and higher bradykinesia severity was observed, albeit this did not reach statistical significance (r = -0.327, *p* = 0.163). Notably, unlike the retrospective cohort, the association between pulse width and Bradykinesia severity was not evident (rho = –0.073, *p* = 0.832). Due to only two male participants in Cohort II, a sex-based comparison was not feasible. The small size of Cohort II precluded robust multivariate modeling.

Finally, we explored the impact of stimulation-induced bradykinesia on quality of life and functional disability using EQ-5D-3L, TWSTRS Disability, and MDS-UPDRS II scales. Despite thorough analysis, no significant associations emerged, suggesting that while bradykinetic symptoms were objectively measurable and clinically observable, they did not significantly influence perceived quality of life or disability levels in this cohort. These negative results are not displayed separately.

### Kinematic analyses

**Fig. 2:**
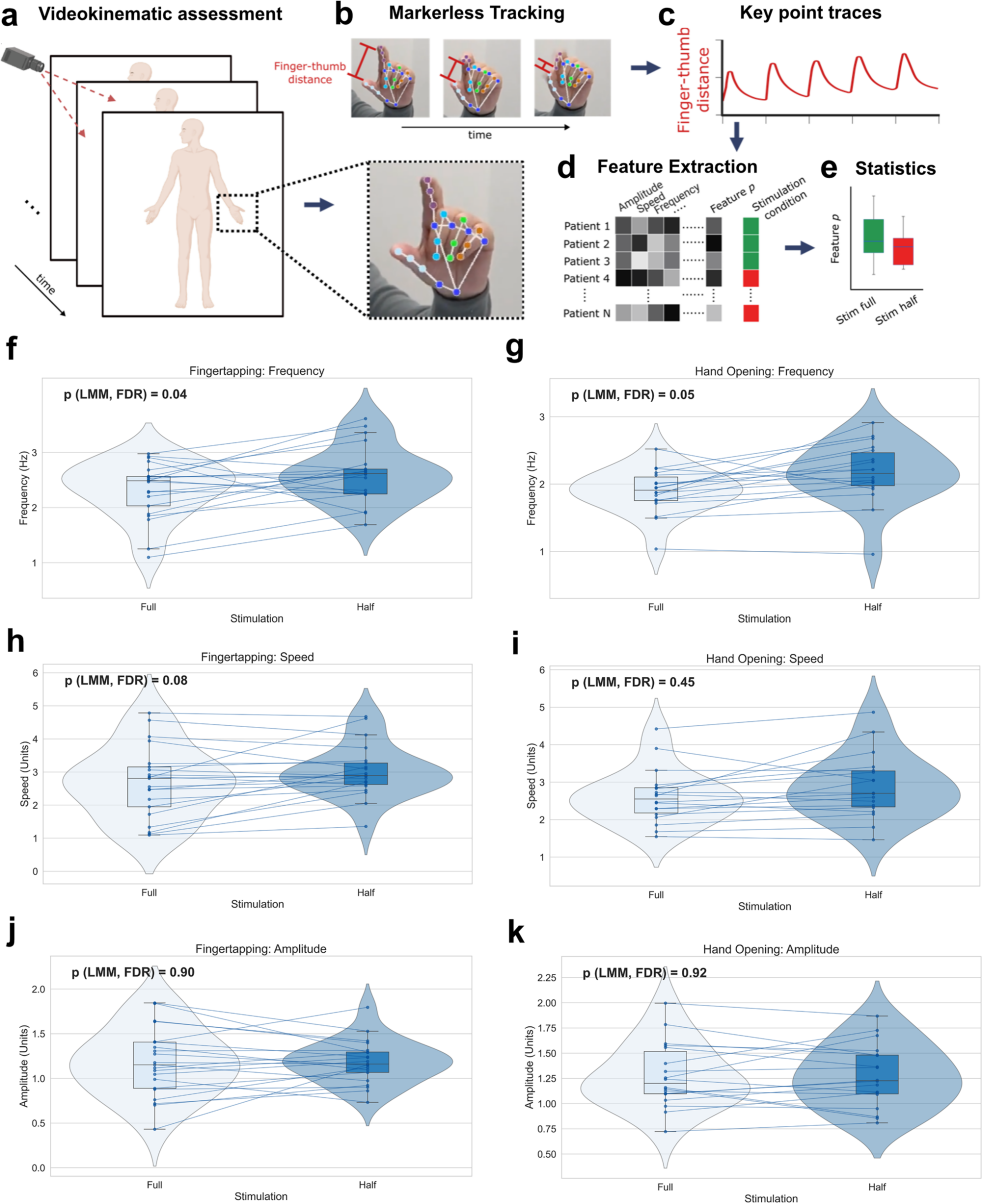
Markerless kinematic analyses of hand movements using the Mediapipe Machine-Learning toolbox. **a** Participants perform structured motor tasks (finger tapping, hand opening) recorded via standardized video protocols. **b** Recorded videos were analysed by Mediapipe, a neural network-based algorithm, precisely tracking hand joints and segments. **c** Kinematic features, such as finger-thumb distance during finger-tapping, were dynamically quantified. **d** Aggregated features, including movement speed, frequency, amplitude, and decay parameters, were extracted per patient and condition and (**e**) used for statistical analysis. **f**, **g** Movement frequency significantly increased under reduced stimulation for finger tapping (*p* = 0.04) and hand opening (*p* = 0.05). **h**, **i** Finger tapping speed improved under reduced stimulation (*p* = 0.08, ns), without clear trend for hand-opening speed showed. **j**, **k** Movement amplitude remained unchanged for both tasks (finger tapping: *p* = 0.888, ns; hand opening: *p* = 0.929, ns).

To objectively quantify stimulation-induced motor changes, we employed a markerless video-based kinematic analysis using the Mediapipe machine-learning toolbox. We specifically assessed two validated motor tasks—finger tapping and hand opening—focusing on movement aspects that indicate bradykinesia: frequency, speed, amplitude, amplitude decay, and velocity decay. Representative methodological steps and results are illustrated in Fig. 2.

At the group level, reducing the amplitude of GPi-DBS led to significant improvements in kinematic parameters indicative of reduced bradykinesia: Movement frequency increased during both finger tapping (from 2.32 ± 0.51 Hz to 2.59 ± 0.51 Hz; estimated increase of 0.27 Hz, *p* = 0.044; Fig. 2f) and hand opening tasks (from 1.94 ± 0.35 Hz to 2.16 ± 0.44 Hz; estimated increase of 0.24 Hz, *p* = 0.048; Fig. 2g) under the reduced stimulation condition. Similarly, movement speed showed a trend towards improvement for both finger tapping (from 2.68 ± 1.08 units to 3.01 ± 0.78 units; p = 0.082; Fig. 2h) and hand opening (from 2.55 ± 0.72 units to 2.88 ± 0.84 units; *p* = 0.454; Fig. 2i), though these changes did not reach statistical significance after correction for multiple comparisons.

In contrast, movement amplitude remained stable across both stimulation condition for finger tapping (*p* = 0.9; Fig. 2j) and hand opening (*p* = 0.9; Fig. 2k). Similarly, we detected no significant differences in amplitude decay nor velocity decay between the two conditions. Given our prior validation of this kinematic analysis pipeline’s sensitivity to subtle changes, the observed absence of differences in amplitude and decay parameters strongly suggests genuine physiological stability rather than measurement insensitivity^15,16^.

Additionally, individual analyses revealed clear signs of bradykinesia—defined by reductions in movement frequency or speed combined with an increased amplitude decay or velocity decay—in 8 out of 11 patients (patients 003, 004, 005, 007, 008, 009, 010, 011) under full stimulation conditions.

To validate our objective kinematic measures against standard clinical assessment, we investigated the relationship between the corresponding MDS-UPDRS III item scores and the kinematic parameters under the full-stimulation condition. For the hand-opening task, higher (worse) clinical scores (item 3.5) correlated negatively with movement frequency (ρ = –0.719, *p* = 0.013) and speed (ρ = –0.563, *p* = 0.071). For the finger-tapping task, higher scores (item 3.4) showed strong inverse correlations with tapping speed (ρ = –0.917, *p* < 0.001) and amplitude (ρ = –0.788, *p* = 0.004), while other parameters showed weaker, non-significant trends in the expected direction. In contrast, and consistent with our primary kinematic findings, no significant correlations were observed between clinical ratings and measures of amplitude decay or velocity decay. The full correlation results are provided in Supplementary Table 2.

### Anatomical mapping of stimulation-induced bradykinesia

**Fig. 3:**
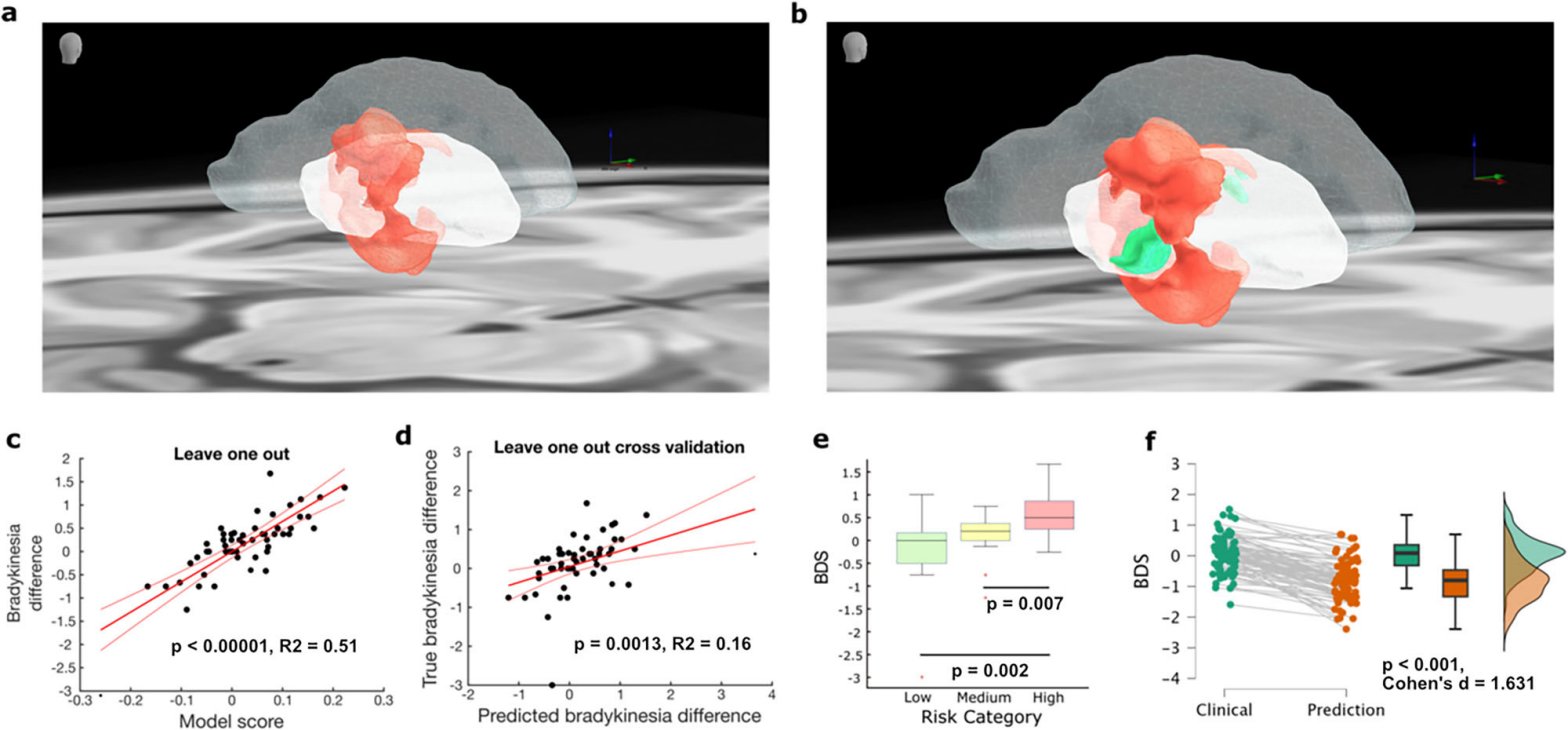
Probabilistic anatomical mapping and clinical risk stratification analyses for stimulation-induced bradykinesia following GPi-DBS for dystonia. **a** Probabilistic “sour spot” map illustrating GPi regions significantly associated with elevated risk (red), based on retrospective cohort voxel-wise analyses. **b** Combined probabilistic map integrating the bradykinesia “sour spot” (red) and dystonia symptom control “sweet spot” (green), highlighting critical areas of overlap and divergence for optimal clinical DBS programming. **c** A linear model based on probabilistic map features (derived using a leave-one-out principle in map generation) demonstrated a strong fit in explaining bradykinesia severity, indicating robust internal consistency of the spatial associations (R² = 0.51, *p* < 0.00001). **d** Patient-level leave-one-out cross-validation demonstrates clinically relevant predictive power for bradykinesia severity (correlation between predicted and actual severities R² = 0.16, *p* = 0.0013). **e** Box plots displaying significant differences among stratified patient risk groups (low, medium, high), particularly prominent between low vs. high (*p* = 0.0019) and medium vs. high-risk patients (*p* = 0.0067). **f** Computational simulations show that machine-assisted reprogramming of DBS settings, guided by the probabilistic risk stratification maps, could theoretically reduce stimulation-induced bradykinesia risk while preserving dystonia motor symptom control (*p* < 0.001).

To identify specific anatomical regions associated with stimulation-induced bradykinesia, we constructed a voxel-wise probabilistic “sour spot” map within the GPi based on the retrospective cohort (Fig. 3a). This map highlights GPi regions linked to an elevated risk of developing Parkinsonian motor symptoms following DBS.

To assess the internal consistency and explanatory power of the identified spatial associations, predictors derived from the probabilistic map for each patient (using a leave-one-out approach in the underlying heatmap data generation to avoid feature bias) were used in a linear model to explain bradykinesia severity. This model demonstrated a strong fit (R² = 0.51, *p* < 0.00001; Fig. 3c), indicating robust internal consistency of the identified spatial associations linked to bradykinesia severity.

Subsequently, to assess the clinical applicability and generalizability of our probabilistic mapping approach, we performed a patient-level leave-one-out cross-validation (LOOCV). In this procedure, each patient’s bradykinesia severity was predicted using a probabilistic map derived from all other patients, reflecting realistic clinical predictive power. This validation confirmed a significant correlation between predicted and observed bradykinesia severity (R² = 0.16, *p* = 0.0013; Fig. 3d).

Using the patient-level LOOCV results, we stratified patients into three distinct risk categories—low, medium, and high—for stimulation-induced bradykinesia (Fig. 3e). This categorization yielded significant differences in bradykinesia severity, especially pronounced when comparing low- vs. high-risk (*p* = 0.0019) and medium- vs. high-risk (*p* = 0.0067, Fig. 3e).

We further examined potential DBS parameter optimization strategies by integrating this newly developed bradykinesia risk map with a previously established GPi dystonia control map for optimal dystonia symptom control^17^ (Fig. 3b). A crucial question for clinical practice is whether the anatomical substrates for therapeutic benefit and side effects are overlapping or distinct. To address this quantitatively, we computed the spatial overlap between the dystonia ‘sweet spot’ and the bradykinesia ‘sour spot’ using a Dice similarity coefficient. The analysis revealed a Dice coefficient of 0.00038, indicating a striking spatial segregation with negligible overlap between the two regions. This finding strongly suggests that stimulation-induced bradykinesia and dystonia symptom improvement are mediated by anatomically distinct populations of neurons or pathways within the pallidal territory. This spatial separation is clinically highly significant, as it implies that through precise electrode placement and programming, it should be possible to selectively modulate the therapeutic zone while actively avoiding the area that produces bradykinetic side effects.

Our computational simulations support this therapeutic potential. As shown in Fig. 3f, by guiding reprogramming based on these two distinct maps, we identified alternative DBS settings for high-risk patients that were predicted to significantly reduce the risk of bradykinesia without compromising dystonia symptom control (*p* < 0.001, Cohen’s d: 1.63).

To further explore potential neuroanatomical substrates underlying stimulation-induced bradykinesia, we analyzed structural connectivity by intersecting patient-specific VTAs with 28 neural pathways from the Peterson connectome^18^ and performed additional verification for the pyramidal tract with the Yeh connectome^19^. No significant correlations between fiber tract activations and bradykinesia were identified, even after incorporating individualized diffusion tensor imaging (DTI) data available for a subset of participants of the prospective cohort (4/11 patients).

Together, these negative connectivity findings strongly suggest that stimulation-induced bradykinesia is unlikely to result from direct stimulation of adjacent white matter fiber tracts. Our data instead point towards a localised disruption within basal ganglia gray matter structures, particularly the GPi as the primary source of bradykinetic side effects.

## Discussion

In the present study, we systematically characterized stimulation-induced bradykinesia in dystonia patients treated with GPi-DBS, identifying key demographic, clinical, and stimulation parameters associated with this unintended stimulation effect. Notably, we found a striking sex disparity, with female patients exhibiting a significantly higher prevalence of stimulation-induced bradykinesia compared to males. Additionally, older age at dystonia onset, shorter disease duration prior to DBS surgery, and higher stimulation pulse widths emerged as strong predictive factors for developing Parkinsonian motor impairments. Prospective kinematic analyses further confirmed the presence of bradykinesia in at least one extremity in 8 out of 11 patients, demonstrating measurable improvements in motor frequency and speed upon reducing stimulation amplitude by 50%. Our probabilistic mapping strongly associated stimulation-induced bradykinesia with modulation of specific GPi gray matter regions. Our extensive analysis suggests that these effects are not caused by a coactivation of adjacent large fiber pathway like the pyramidal tract.

The term ‘bradykinesia’, originally described by James Parkinson as “lessened muscular power”, has evolved significantly and now broadly encompasses hypokinesia (reduced movement amplitude), akinesia (absence of movement), and the ‘sequence effect’—a progressive decrement in speed or amplitude with repetition^20,21^. Although these phenomena often coexist in Parkinson’s disease, recent evidence suggests these motor deficits reflect distinct pathological mechanisms. For instance, levodopa improves bradykinesia and hypokinesia but not the sequence effect^22–25^, and the sequence effect may be absent in Progressive Supranuclear Palsy compared to PD and Multiple System Atrophy^26,27^.

Our prospective kinematic assessments, employing a sensitive, video-based machine-learning approach, provides robust quantitative evidence confirming the classic features of bradykinesia under full stimulation conditions—specifically, reductions in movement speed and frequency. Importantly, while 8 out of 11 patients met the Movement Disorders Society and EFNS criteria for bradykinesia (requiring slowness and a decrement in amplitude or speed during continued movement)^28,29^, we did not observe significant concurrent reductions in amplitude, amplitude decay, or velocity decay (notably all 11 patients had non-zero MDS-UPDRS III bradykinesia scores across all items, so clinical signs of bradykinesia in all patients).This distinct profile of isolated slowing without amplitude impairment marks the possibility of independent neural circuits governing movement speed and amplitude control within the GPi^22,30–32^.

Several models have proposed that the basal ganglia act as a “clearing house” that filter out unwanted movements while selecting appropriate actions^33,34^. We hypothesize that within the GPi, distinct functional networks selectively modulate motor speed and amplitude. Under this “Network Specificity” hypothesis, GPi-DBS preferentially interferes with circuits governing movement speed—potentially involving differential modulation of direct, indirect, or hyperdirect cortico-subthalamic pathways—while leaving amplitude-regulatory circuits comparatively unaffected. This hypothesis is supported by recent neurophysiological evidence from Lofredi et al., who demonstrated that DBS-induced slowness in dystonia patients is directly mirrored by an increase in pallidal low-beta band (13-20 Hz) oscillations^12^. This finding provides a direct physiological correlate for the bradykinesia we observed and suggests that the ‘sour spot’ identified in our anatomical mapping may correspond to a GPi subregion where stimulation is particularly effective at inducing or potentiating this pathological beta activity. As noted by Bergman, “the indirect pathway supplies an inhibitory blanket in the GPi, out of which the direct pathway carves the intended movement in a winner-takes-all dynamic, helping ensure that a single action is selected”^34^.

An alternative hypothesis, the “Sequential Functional Breakdown” model, suggests a staged impairment in GPi function, with initial disruptions to speed-regulating networks manifesting prior to impairment of amplitude control. This sequential model would be consistent with clinical observations that optimal dystonic symptom relief via GPi-DBS can take weeks to months^3,35^, potentially reflecting delayed compensatory adjustments within basal ganglia circuits. We hypothesize that the initial disruption of speed-regulating networks (manifesting as bradykinesia) could represent the most immediate stage of this broader network modulation, potentially preceding impacts on more resilient amplitude-control circuits. Our results are consistent with both hypotheses but those need further experimental validation.

Our investigation identified several demographic and clinical factors increasing the risk of stimulation-induced bradykinesia following GPi-DBS for dystonia. Most notably, we found a marked sex disparity: female patients exhibited significantly greater susceptibility (Odds Ratio 4.66, p < 0.008). This finding is particularly interesting given that the role of sex in DBS outcomes remains a subject of debate. For instance, while some reports have suggested poorer outcomes for women with advanced PD treated with dopaminergic therapy or STN-DBS^36,37^, other studies have not found significant sex-based differences.

It is important to distinguish these univariate associations from independent predictors. In addition to sex, our univariate analyses also showed that older age at dystonia onset and shorter disease duration were significantly correlated with bradykinesia severity. Yet, when all these factors were included in our final multivariate model, none of them—sex, age of onset, or disease duration—emerged as significant independent predictors after accounting for stimulation parameters. This suggests that while these demographic factors are associated with risk, their predictive power may be linked to other underlying variables or not be strong enough to independently predict outcomes once stimulation settings are considered.

The potential reasons for these associations, particularly the marked sex disparity, remain unknown, possible contributors include known sexual dimorphism in basal ganglia physiology and neurochemistry, potentially modulated by sex hormones like estrogen^38–44^.

Furthermore, univariate analyses show that older age at dystonia onset (rho = 0.329, *p* = 0.016) and shorter disease duration prior to surgery (rho = –0.379, *p* = 0.005) were associated with increased bradykinesia severity upon DBS. Importantly, the severity measure in this retrospective cohort, the Bradykinesia Difference Score (BDS), reflects the change from pre- to post-operative assessments. This calculation inherently helps control for simple baseline motor slowing related to the patient’s chronological age before surgery. However, it cannot fully exclude the possibility that age influences the susceptibility of the motor system to the disruptive effects of DBS. Despite the partial mitigation offered by the BDS, neither age of onset nor disease duration emerged as significant independent predictors in our final multivariate model after accounting for stimulation parameters (particularly pulse width) and other covariates. While these age-related factors have previously been linked to less favorable dystonia symptom control following GPi-DBS^1,35,45–48^ their lack of independent prediction for bradykinesia in our model limit interpretations about shared vulnerability based solely on these demographic factors.

A plausible explanation for the univariate associations in our two independent cohorts may still involve age-related neurobiological changes. Younger individuals may possess greater neuroplasticity, facilitating adaptation to stimulation-induced neuronal activity patterns^49,50^ Conversely, older adults often exhibit reduced basal ganglia functional reserve and adaptability^51^, compounded by age-related declines in nigral dopamine neurons and striatal D2/3 receptor availability^52–56^. This diminished capacity could potentially render the aging basal ganglia network more susceptible to disruption by DBS, manifesting as side effects like bradykinesia, even if this effect was not independently significant after accounting for stimulation parameters in our analysis.

Prior studies have frequently attributed DBS-induced bradykinesia to electrode deviations along the along the anterior-posterior and superior-inferior axes^4,13,57,58^. However, our probabilistic mapping challenges this notion, indicating instead that stimulation-induced bradykinesia risk rises circumferentially when the VTA deviates broadly from the optimal GPi “sweet spot” particularly along superior–inferior and medial–lateral axes (Fig. 3b). Rigorous cross-validation of our probabilistic model demonstrates strong predictive accuracy at both voxel and patient levels, effectively stratifying patients into clinically meaningful risk categories based on the distribution of stimulated tissue.

Our probabilistic map demonstrates strong internal consistency at the voxel level (R² = 0.51; Fig. 3c), robustly identifying the anatomical substrate associated with bradykinesia. The patient-level cross-validation yielded a more modest, though statistically significant, predictive power for individual clinical severity (R² = 0.16; Fig. 3d). This significant patient-level correlation confirms the map’s relevance and its utility for effective risk stratification (Fig. 3e) and guiding DBS programming adjustments aimed at mitigating bradykinesia (Fig. 3f).

Our comprehensive exploration of structural connectivity involving normative and for four cases patient-specific connectomes reveals no significant correlations between white-matter fiber activation and bradykinesia occurrence. These findings challenge the hypothesis that stimulation-induced bradykinesia is primarily mediated by the activation of specific white-matter pathways, such as the frequently implicated pyramidal tract. Our data suggest these symptoms are more likely to arise from disruption of GPi gray-matter. This conclusion is consistent with previous work which, using EMG-based assessment, also failed to establish a link between functional proximity to the pyramidal tract and parkinsonian signs^9^.

Some version of stimulation-induced parkinsonism was frequently encountered with GPi-DBS for dystonia, observed in 60% of our retrospective cohort and suggested by kinematic slowing in 72% (8/11) of our prospective group. While selection bias may influence retrospective estimates, these high rates, also reported in non-dystonia DBS^59,60^, highlight the clinical relevance of PD-like side effects and potential for underdiagnosis. Although our prospective analysis did not link these motor side effects to diminished quality of life (possibly due to limited cohort size or symptom severity) literature and clinical experience confirm that severe, debilitating parkinsonism can occur as a result of DBS^10^, necessitating effective therapeutic strategies.

Our study identified stimulation pulse width as a strong, independent predictor of bradykinesia severity (*p* = 0.004, GLM analysis) in Cohort I. This association could not be reproduced in Cohort II, but this non-finding should be interpreted with caution, as it may be due to the narrower range and overall lower mean pulse width in the prospective Cohort II (82.7 µs ± 25.3) compared to the retrospective Cohort I (112 µs ± 43), eliminating the impact of large pulse widths ( > 120 µs) and potentially limiting sensitivity to detect the relationship. Furthermore, this contrast invites speculation about a potential threshold effect, where the risk of bradykinesia might escalate more steeply only when pulse widths exceed a certain duration (e.g., >120 µs), a range less frequently encountered in Cohort II.

The strong association of bradykinesia severity with pulse width in Cohort I stands in strong contrast with the lack of correlation observed for stimulation amplitude. This dissociation suggests pulse width may exert a differential recruitment of neuronal elements with varying chronaxies^61^. Furthermore, the absence of a clear link between dystonia symptom control and bradykinesia occurrence in our data favors the hypothesis of distinct underlying mechanisms rather than a simple increase in the volume of tissue activated.

Therefore, alongside optimizing electrode placement using tools like probabilistic mapping^62–65^, our findings strongly advocate for considering pulse width reduction as a primary strategy to mitigate GPi-DBS-induced bradykinesia in dystonia patients.

While our study provides clinically relevant new information into stimulation-induced bradykinesia in dystonia patients undergoing GPi-DBS, several limitations should be acknowledged.

First, the retrospective design inherently limits our ability to draw causal inferences between the examined factors and the occurrence of bradykinesia. Future longitudinal studies that monitor patients both pre- and post-DBS could offer a more comprehensive understanding of these relationships.

The retrospective multicenter cohort introduces additional heterogeneity. Variability in video acquisition protocols, rating scales, and DBS programming practices across centers may affect the consistency and reliability of the assessments. To handle this heterogeneity, we developed study-specific metrics (NBS and BDS) for normalizing bradykinesia scores. It is important to note that these novel scores, while designed for this purpose, have not undergone formal validation for reliability and validity, which should be considered when interpreting the results from Cohort I.

Similarly, while our prospective analysis allowed for in-depth kinematic evaluation, the small sample size (*N* = 11) restricts the generalizability of these findings. This modest sample size, along with a significant sex imbalance (9 females, 2 males) and a narrow range of stimulation parameters, limits the statistical power required to detect potentially more subtle kinematic effects. Consequently, this cohort was unsuited for validating the demographic and programming-related risk factors identified in Cohort I, such as the roles of sex and pulse width. This also precluded the application of complex multivariate analyses within this cohort to explore interactions between variables. Furthermore, while Cohort II provided valuable kinematic data, its small sample size and experimental design—which intentionally varied stimulation amplitude—rendered it unsuitable for validating the anatomical ‘sour spot’ map derived from Cohort I. Such a validation would be confounded by VTA size and requires a separate, adequately powered prospective study.

Our use of markerless 2D video tracking via the MediaPipe toolbox provides objective kinematic data, yet limitations inherent in 2D analysis warrant discussion. While standardized recording protocols and normalization were employed, the lack of depth information remains a factor. This is particularly relevant to the observed kinematic dissociation where movement speed and frequency improved upon reducing stimulation, while amplitude remained stable. Movements with a significant component out of the primary analysis plane could potentially lead to underestimations of true 3D speed and amplitude. Although normalization mitigates size scaling issues, we acknowledge the possibility that such 2D projection effects might subtly influence the relative sensitivity of detecting changes in speed versus amplitude compared to a full 3D analysis.

Furthermore, our multivariate model explained approximately 22% of the variance in bradykinesia severity. While this confirms the statistical and clinical importance of the identified factors, it also underscores that a large portion of the variance remains unaccounted for. This strongly suggests that other variables—such as individual genetic predispositions, sub-clinical network differences, or microstructural tissue properties not captured in this study—likely play a crucial role and warrant dedicated future investigation.

In our connectivity analyses, we primarily relied on normative connectomes to assess the intersection of volume-activated tissues (VTAs) with neural pathways. While this approach provides a useful reference, it may not fully capture individual neuroanatomical variability. Importantly, the conclusion that bradykinesia is not mediated by white matter tracts is based on a negative finding. The absence of evidence for an association with the investigated tracts is not definitive evidence of absence, as the involvement of smaller, unmapped pathways or inter-individual variations cannot be entirely ruled out. Although a patient-specific DTI dataset from four patients was available and used to validate our findings, its small size limits broader conclusions regarding structural connectivity.

In conclusion, our study identifies several clinical risk factors associated with stimulation-induced bradykinesia following GPi-DBS, including female sex, older age at dystonia onset, and shorter disease duration. Critically, we demonstrate that higher stimulation pulse width is a significant and independent predictor of bradykinesia severity, suggesting pulse width as the primary parameter for reprogramming. Furthermore, prospective kinematic analysis revealed a distinct motor signature (reduced movement frequency sensitive to stimulation changes, without concomitant movement amplitude reduction) suggesting selective disruption of specific pallidal circuits. Our probabilistic mapping could provide a predictive tool for risk stratification, which can be used for optimizing DBS programming strategies in the future.

## Methods

**Fig. 4:**
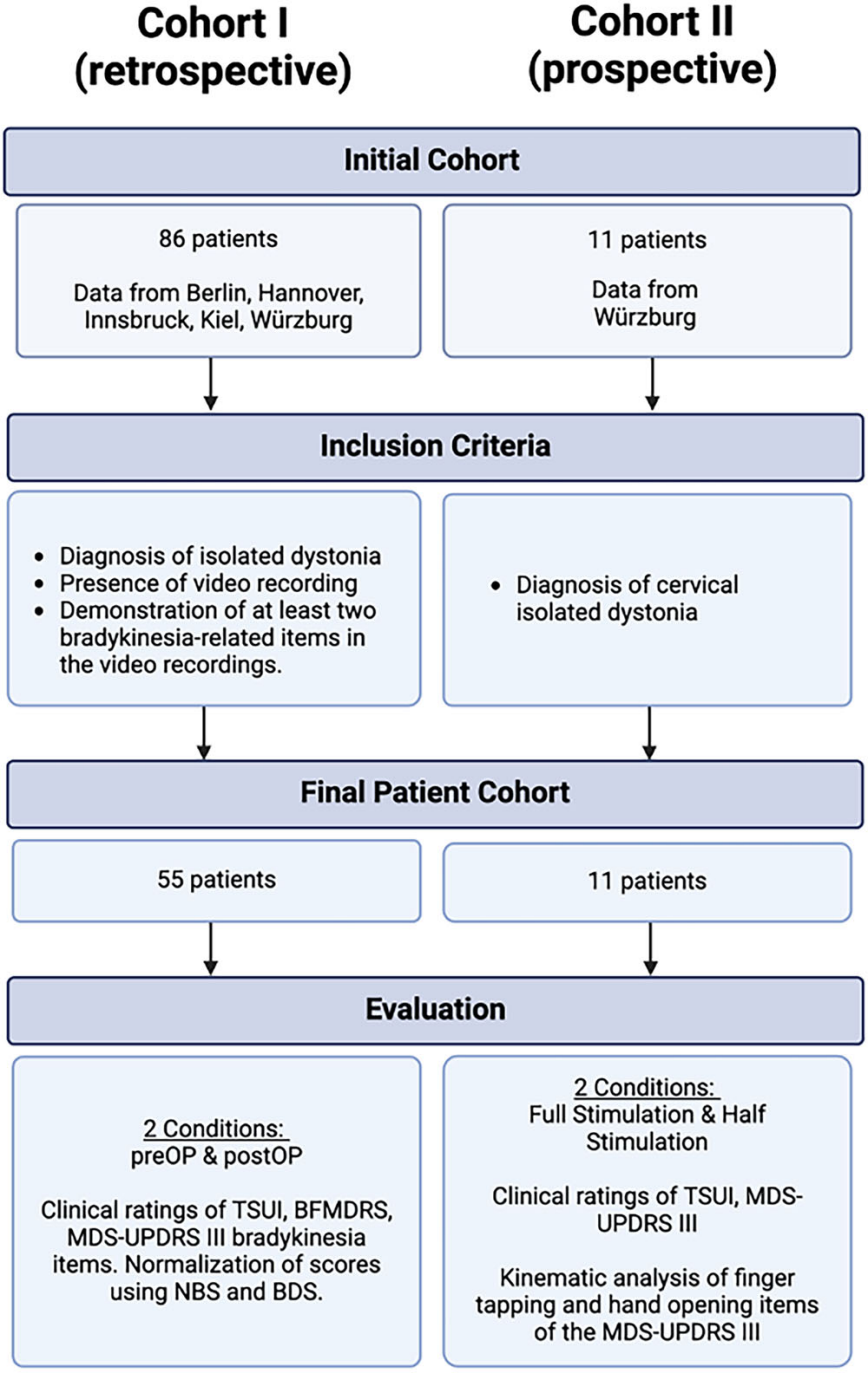
Flowchart of study design. DBS Deep Brain Stimulation, TSUI Tsui Dystonia Rating Scale, MDS-UPDRS Movement Disorder Unified Parkinson’s Disease Rating Scale, NBS Normalized Bradykinesia-Score, BDS Bradykinesia Difference Score, MDS-UPDRS III Movement Disorder Unified Parkinson’s Disease Rating Scale Part III.

We conducted two complementary studies to investigate the impact of GPi-DBS on dystonia patients, with a focus on stimulation-induced parkinsonism. A multicenter retrospective study (Cohort I) was designed to identify risk factors, while a single-center prospective trial (Cohort II) examined the kinematic features and their effects on the quality of life. In both cohorts, we collected video sequences to evaluate symptom control and stimulation-induced side effects. For Cohort II, a more extensive half-day evaluation was carried out, incorporating both qualitative and quantitative analyses. The study flow is illustrated in Fig. 4. The study was conducted in compliance with the Declaration of Helsinki and received approval from the institutional review board of the University Hospital of Würzburg (registration no. 150/15).

### Cohort I: retrospective study

For Cohort I, we reviewed the records of 86 adult dystonia patients from various DBS centers who had undergone chronic bilateral pallidal DBS. Inclusion criteria for this cohort consisted of a prior diagnosis of isolated generalized, segmental, or cervical dystonia, availability of pre- and postoperative video assessments, and demonstration of at least two items of the Movement Disorder Unified Parkinson’s Disease Rating Scale (MDS-UPDRS-III) that evaluate limb involvement (such as finger tapping, wrist grasping, pronation/supination, toe tapping, or leg agility, as described by the Movement Disorder Unified Parkinson’s Disease Rating Scale; items 3.4, 3.5, 3.6, 3.7, 3.8) on both pre- and post-surgery videos. To identify potential preoperative predictors of stimulation-induced bradykinesia, we evaluated demographic and baseline clinical features, such as age at surgery, sex, genetics, age at dystonia onset, disease duration, and the effectiveness of DBS treatment on dystonic motor symptoms. This cohort was previously used for constructing a probabilistic outcome map of pallidal DBS for dystonia^17,66^.

### Cohort II: prospective study

For the prospective cohort, we invited all patients diagnosed with isolated cervical dystonia or segmental dystonia with cervical focus (defined as dystonia affecting the neck and at least one contiguous body region) who attended our outpatient clinic during the designated study enrollment period (01/2020–12/2020). Eligibility for the trial was determined without consideration of the patients’ level of dystonia symptom control or any side effects experienced prior to screening. This approach was intended to capture a representative sample of the cervical dystonia patient population seen in our clinic. Exclusion criteria were the presence of any concurrent diseases or any dystonic features in the limbs to not confound the clinical and kinematic evaluation. Patients in this cohort were assessed in two separate stimulation settings: their chronic settings, stable for over three months, and modified settings with stimulation amplitudes reduced by 50% after 60 min of wash-out. The evaluations, covering a half-day of neurological examinations, included BFMDRS, TSUI, and MDS-UPDRS III scales, as well as specific assessments targeted to identify disturbances in speech, gait and handwriting.

### Clinical evaluation

We obtained preoperative and postoperative videos for each patient in the retrospective cohort, as well as videos under full and half stimulation conditions for the prospective cohort. For patients with cervical/segmental dystonia, we used the Tsui Dystonia Rating Scale (TSUI) to evaluate symptom control. For those with generalized dystonia, the Burke-Fahn-Marsden Dystonia Rating Scale (BFMDRS) was applied. Additionally, we assessed stimulation side effects using the Movement Disorder Society’s Unified Parkinson’s Disease Rating Scale (MDS-UPDRS III). Trained movement disorder neurologists (C.D., G.B., T.O.), blinded to the stimulation conditions, independently rated each one of the videos.

For the retrospective data set, we used on an aggregation of videos from different DBS centers, which did not adhere to a prespecified protocol, and the MDS-UPDRS III, a Parkinson-specific scale, was not used in its entirety in any of the videos. However, this scale is commonly utilized to identify features indicative of non-isolated dystonia, and as such, several items were present in most videos that could be employed to measure parkinsonian-like symptoms. Owing to the retrospective nature of the analysis and the difficulty in distinguishing bradykinesia features in the presence of severe dystonia, items were omitted in limbs severely affected by dystonia.

To facilitate comparison between patients and account for the varying number of bradykinesia-related MDS-UPDRS III items available in the retrospective video recordings, we devised a study-specific Normalized Bradykinesia-Score (NBS). This score normalizes the sum of the item ratings to the number of items assessed for each individual patient, allowing for robust analysis despite the heterogeneous data. The NBS was obtained by the following formula:

1 $${NBS}=\frac{\sum \left({Subitem\; Scores}\right)}{{Number\; of\; Subitems\; Used}}$$

where ∑ (Subitem Scores) represents the sum of scores for all MDS-UPDRS III subitems included in the video assessments, and Number of Subitems Used is the total count of these subitems. Subsequently, we derived a Bradykinesia Difference Score (BDS) to quantify changes in bradykinetic features postoperatively:

2 $${BDS}={NBS}\mathrm{post}-{NBS}\mathrm{pre}$$

In this equation NBSpre is the Normalized Bradykinesia-Score before surgery, and NBSpost is the Normalized Bradykinesia-Score after surgery. This comparison of normalized scores provides a robust measure of change even when the specific subitems available for rating differed between a patient’s pre- and postoperative recordings. A BDS value greater than 0 indicates a postoperative worsening in parkinsonian-like symptoms.

For the prospective trial, patients with cervical dystonia were assessed with an extensive neurological examination including the items of TSUI score and MDS-UPDRS III in two sessions, first with their chronic, full-stimulation amplitude and after 60 min in half amplitude stimulation. All examinations were video-recorded. This captured footage was used in a dual-layered analytical approach: Firstly, a qualitative evaluation conducted by movement disorder specialists (as described above), and secondly, a quantitative evaluation using a kinematic machine-learning algorithm to delineate specific movement variables. Subjective assessment of gait disturbances was evaluated with the Freezing of Gait Questionnaire (FOGQ) and effects on quality of life was assessed by the EQ-5D-3L score.

Importantly, all patients were classified as isolated dystonia patients at the time of DBS implantation.

### Markerless kinematics

We developed a semiautomatic videokinematic analysis pipeline, VisionMD, for tracking movements in neurological disorders, enabling the precise tracking of complex hand motions without the need for physical markers (see Code Availability and^67^; the latest version and further details are available at https://visionmd.ai/). In this project, we measured key indices of bradykinesia: movement frequency, amplitude, speed, amplitude decay, and velocity decay of two hand movements commonly used to assess Parkinsonian features: rapidly alternating finger tapping and hand opening (MDS-UPDRS 3.4 and 3.5).

For the finger tapping task, we calculated the distance between the thumb and index fingertips. For the hand opening assessment we measured the angles formed by three points: the wrist, the metacarpophalangeal joint, and the fingertip, for each of the four fingers (four angles per hand). This analysis was conducted for every frame of the movement sequence, allowing us to derive not only amplitude but also temporal parameters such as the duration of individual movement cycles and their decay over time. Thus, we were capturing both static postures and dynamic aspects of the movements.

A critical consideration arises when comparing the amplitude of movements due to the inherent problem of depth perception in 2D video analysis. This limitation can lead to disparities in amplitude measurements due to perspective distortion. To address this, we normalized the movements to the size of the full hand measured as the distance from the metacarpophalangeal joint of the middle finger to the base of the palm for the hand opening task and to the length of the thumb for the finger tapping task. All amplitude measurements were then scaled proportionally to this maximum value.

By recording these metrics frame-by-frame throughout the videos, we generated a time-series dataset that chronicles the progression of hand kinematics, providing a rater-independent, objective measure of motor function. This process is illustrated in Fig. 2. The most recent version of this videokinematic toolbox is updated at https://visionmd.ai/.

### Methodological framework for analyzing VTA effects on DBS-induced side effects

To analyze the effects of the volume of tissue activation (VTA) on the observed side effects, we used a similar pipeline as described previously to analyze effects on dystonia symptom control^17^. Briefly, the visualization of electrode positions was established using SureTune™ software (Medtronic Eindhoven Design Center, MEDC) by fusing the individual preoperative MRIs used for stereotactic planning with postoperative CT or MRI images that depicted the lead location. The electrode locations were identified by the CT or MRI artifacts, consistent with established protocols^68,69^, and mapped onto the Yelnik atlas^70^. The atlas was registered to key anatomical regions (GPi, GPe, striatum) using a rigid semi-automatic registration for each hemisphere independently. The VTA for each lead was computed based on the applied stimulation parameters, using the method described by Astrom et al. ^71^. This biophysical model generates patient-specific VTAs by integrating the effects of amplitude, pulse width, and active contact configuration, meaning that the VTA itself already accounts for the influence of these programming settings. VTAs were then linearly normalized into the Montreal Neurological Institute (MNI; ICBM 2009b NLIN asymmetric) space.

### Creation of volume of tissue activated-based outcome maps for Bradykinesia and risk stratification of Bradykinesia

After the special normalization we determined the VTAs overlapping each voxel and conducted a voxelwise two-sample t-test of motor control between patients with stimulation at that voxel and patients without. This resulted in 3D statistical maps with t-statistics and *P* value for each voxel. Clusters comprising more than 500 voxels with an uncorrected *p* value below 0.05 were considered significant. Based on the t-statistic direction, these clusters were identified as regions significantly associated with the induction of stimulation-induced bradykinesia. This “sour spot” was then used to overlay individual patient VTAs, enabling a quantification of the bradykinesia risk by calculating the average probability scores within the VTA overlays. Similar approaches have been used in our previous work and are explained in more detail in the respective original publications for mapping of symptom control in dystonia^17^ and evaluating risks of cognitive decline in STN-DBS^72^.

### Connectomic analyses

The connectomic analyses were conducted using a custom-built software platform, ARENA, specifically developed for integrating and analyzing neuroimaging data in Deep Brain Stimulation (DBS) research [https://github.com/visualDBSlab/ARENA].

The following fibers were imported from the connectome developed by Petersen et al. ^18^ which reconstructs human brain axonal pathways by integrating histological and MRI data: ansa lenticularis, cerebellothalamic, Gpe to STN associative, Gpe to STN sensorimotor, lenticular fasciculus, medial lemniscus, STN to Gpe associative, STN to Gpe sensorimotor, STN to GPi associative, STN to GPi sensorimotor, M1 corticofugal face, M1 corticofugal lower extremity, M1 corticofugal upper extremity, M1 hyperdirect pathway face, M1 hyperdirect pathway lower extremity, M1 hyperdirect pathway upper extremity, premotor corticofugal, premotor hyperdirect pathway, SMA corticofugal, SMA hyperdirect pathway, ACC corticopontine, ACC hyperdirect pathway, dlPFC corticopontine, dlPFC hyperdirect pathway, dmPFC corticopontine, vlPFC corticopontine, vmPFC corticopontine, and vmPFC hyperdirect pathway. Given the significant role of the pyramidal tract in motor control and its previously proposed potential involvement in DBS-induced motor abnormalities, we redundantly used another version of the pyramidal tract, extracted from the connectome of Yeh^19^, implemented in DSI Studio. In addition to the normative connectomes, a patient-specific diffusion tensor imaging (DTI) dataset from four patients was available and used to evaluate the intersection analyses of VTAs with neural pathways.

The intersections between the VTAs and the imported fiber tracts were calculated to provide a quantitative estimation of fiber engagement by the activated tissue. Using ARENA’s integrated statistical tools, Spearman’s correlation coefficients and mixed-effect models were calculated to assess the relationship between the intersected fiber counts and the bradykinesia scores.

### Statistical analysis

Statistical analyses were performed using JASP software and Python’s statsmodels. Chi-square tests were employed to assess differences in categorical data. To perform the Chi-square test, we additionally binarized the stimulation-induced bradykinesia outcome, designating it as either present (1) or not present (0). The response of the patient’s dystonia motor symptoms to DBS (Responder Status) was grouped into four response categories: non-responder ( < 25%), average responder (25–50%), good responder (50–80%), and super-responder ( > 80%).

For the retrospective cohort (Cohort I), the correlation between Bradykinesia severity (BDS) and the demographic and baseline clinical features was analyzed using Spearman correlation coefficients for quantitative variables. We then used a Generalized Linear Model (GLM) to evaluate the combined effect of the following predictor variables: age of onset, disease duration, amplitude, Pulse Width, Frequency, Sex, Type of Dystonia and Responder type on the dependent variable (BDS).

For the prospective cohort (Cohort II), we constructed mixed-effects linear models, analyzing the impact of varying stimulation settings on kinematic variables. This analysis accounted for the within-subject correlation across conditions (full and half amplitude stimulation) and included fixed effects for the fiber counts and random effects to handle inter-individual variability.

Statistical significance was set at α = 0.05. Where multiple comparisons were performed, adjustments were made using the False Discovery Rate (FDR) method, and all reported *p* values incorporate these corrections.

## Supplementary information

Supplementary Tables

## Data Availability

The anonymized patient data and the derived kinematic features data matrix used for all statistical analyses is publicly available in this GitHub repository: https://github.com/Flolan2/Bradystim_data.

## Code availability

The core VisionMD computer vision pipeline used for feature extraction is open-source and available for download at https://www.visionmd.ai/. ARENA, which was used for imaging-based analyses like probabilistic is open-source and available for download at https://github.com/visualDBSlab/ARENA.

## References

[1] Vidailhet, M. et al. Bilateral deep-brain stimulation of the globus pallidus in primary generalized dystonia. *N. Engl. J. Med.* **352**, 459–467 (2005). 15689584 10.1056/NEJMoa042187

[2] Vidailhet, M. et al. Bilateral, pallidal, deep-brain stimulation in primary generalised dystonia: a prospective 3 year follow-up study. *Lancet Neurol.* **6**, 223–229 (2007). 17303528 10.1016/S1474-4422(07)70035-2

[3] Volkmann, J. et al. Pallidal deep brain stimulation in patients with primary generalised or segmental dystonia: 5-year follow-up of a randomised trial. *Lancet Neurol.* **11**, 1029–1038 (2012). 23123071 10.1016/S1474-4422(12)70257-0

[4] Berman, B. D., Starr, P. A., Marks, W. J. & Ostrem, J. L. Induction of bradykinesia with pallidal deep brain stimulation in patients with cranial-cervical dystonia. *Stereotact. Funct. Neurosurg.* **87**, 37–44 (2009). 19174619 10.1159/000195718 PMC2835378

[5] Blahak, C. et al. Micrographia induced by pallidal DBS for segmental dystonia: A subtle sign of hypokinesia?. *J. Neural Transm.* **118**, 549–553 (2011). 21246224 10.1007/s00702-010-0544-y

[6] Wolf, M. E. et al. Hypokinetic gait changes induced by bilateral pallidal deep brain stimulation for segmental dystonia. *Gait Posture*. **49**. 10.1016/j.gaitpost.2016.07.301 (2016). 10.1016/j.gaitpost.2016.07.301 27491053

[7] Moscovich, M. Freezing of Gait After Bilateral Globus Pallidus Interna Deep Brain Stimulation in Generalized Dystonia. In: *Deep Brain Stimulation*. 10.1093/med/9780190647209.003.0021 (2020). 10.1002/mdc3.12071 PMC6353365 30713864

[8] Huebl, J. et al. Bradykinesia induced by frequency-specific pallidal stimulation in patients with cervical and segmental dystonia. *Parkinsonism Relat Disord*. 21. 10.1016/j.parkreldis.2015.04.023 (2015). 10.1016/j.parkreldis.2015.04.023 25976986

[9] Mahlknecht, P. et al. Parkinsonian signs in patients with cervical dystonia treated with pallidal deep brain stimulation. *Brain* **141**, 3023–3034 (2018). 30165511 10.1093/brain/awy217

[10] Reese, R. et al. Full parkinsonian triad induced by pallidal high-frequency stimulation in cervical dystonia. *Mov. Disord. Clin. Pr.* **2**, 99 (2015). 10.1002/mdc3.12105 PMC6183174 30363825

[11] Cagle, J. N. et al. Suppression and rebound of pallidal beta power: Observation using a chronic sensing DBS device. *Front Hum. Neurosci*. 15. 10.3389/fnhum.2021.749567 (2021). 10.3389/fnhum.2021.749567 PMC8458625 34566612

[12] Lofredi, R. et al. Pallidal beta activity is linked to stimulation-induced slowness in dystonia. *Mov. Disord.* **38**, 894–899 (2023). 36807626 10.1002/mds.29347

[13] Tisch, S. et al. Effect of electrode contact location on clinical efficacy of pallidal deep brain stimulation in primary generalised dystonia. *J Neurol Neurosurg. Psychiatry.* **78**, 1314–1319 (2007).. 10.1136/jnnp.2006.109694 PMC2095629 17442760

[14] Schrader, C. et al. GPi-DBS may induce a hypokinetic gait disorder with freezing of gait in patients with dystonia. *Neurology* **77**, 483–488 (2011). 21775741 10.1212/WNL.0b013e318227b19e

[15] Trebbau, G. T. A., Bandini, A., Guarin, D. L. Video-Based Hand Pose Estimation for Remote Assessment of Bradykinesia in Parkinson’s Disease. Published online August 28, 2023. Accessed October 24, https://arxiv.org/abs/2308.14679v1 (2024).

[16] Guarín, D. L., Wong, J. K., McFarland, N. R., Ramirez-Zamora, A. & Vaillancourt, D. E. What the trained eye cannot see: Quantitative kinematics and machine learning detect movement deficits in early-stage Parkinson’s disease from videos. *Parkinsonism Relat. Disord.* **127**, 107104 (2024). 39153421 10.1016/j.parkreldis.2024.107104 PMC13501229

[17] Reich, M. M. et al. Probabilistic mapping of the antidystonic effect of pallidal neurostimulation: a multicentre imaging study. *Brain* **142**, 1386–1398 (2019). 30851091 10.1093/brain/awz046

[18] Petersen, M. V. et al. Holographic reconstruction of axonal pathways in the human brain. *Neuron* **104**, 1056–1064.e3 (2019). 31708306 10.1016/j.neuron.2019.09.030 PMC6948195

[19] Yeh, F. C. Population-based tract-to-region connectome of the human brain and its hierarchical topology. *Nat. Commun.* **13**, 4933 (2022). 35995773 10.1038/s41467-022-32595-4 PMC9395399

[20] Berardelli, A., Rothwell, J. C., Thompson, P. D. & Hallett, M. Pathophysiology of bradykinesia in parkinson’s disease. *Brain* **124**, 2131–2146 (2001). 11673316 10.1093/brain/124.11.2131

[21] Parkinson, J. An essay on the shaking palsy. 1817. *J. Neuropsychiatry Clin. Neurosci.* **14**, 223–236 (2002). 11983801 10.1176/jnp.14.2.223

[22] Agostino, R., Berardelli, A., Formica, A., Accornero, N. & Manfredi, M. Sequential arm movements in patients with parkinson’s disease, huntington’s disease and dystonia. *Brain* **115**, 1481–1495 (1992). 1422799 10.1093/brain/115.5.1481

[23] Kang, S. Y. et al. Characteristics of the sequence effect in Parkinson’s disease. *Mov. Disord.* **25**, 2148–2155 (2010). 20669182 10.1002/mds.23251 PMC4782591

[24] Suppa, A., Bologna, M., Conte, A., Berardelli, A. & Fabbrini, G. The effect of L-dopa in Parkinson’s disease as revealed by neurophysiological studies of motor and sensory functions. *Expert Rev. Neurother.* **17**, 181–192 (2017). 27477028 10.1080/14737175.2016.1219251

[25] Wu, T. et al. Neural correlates underlying Micrographia in Parkinson’s disease. *Brain* **139**, 144–160 (2016). 26525918 10.1093/brain/awv319 PMC4719707

[26] Ling, H., Massey, L. A., Lees, A. J., Brown, P. & Day, B. L. Hypokinesia without decrement distinguishes progressive supranuclear palsy from Parkinson’s disease. *Brain* **135**, 1141–1153 (2012). 22396397 10.1093/brain/aws038 PMC3326257

[27] Djurić-Jovičić, M. et al. Finger tapping analysis in patients with Parkinson’s disease and atypical parkinsonism. *J. Clin. Neurosci.* **30**, 49–55 (2016). 27343040 10.1016/j.jocn.2015.10.053

[28] Berg, D. et al. Movement disorder society criteria for clinically established early Parkinson’s disease. *Mov. Disord.* **33**, 1643–1646 (2018). 30145841 10.1002/mds.27431

[29] Postuma, R. B. et al. MDS clinical diagnostic criteria for Parkinson’s disease. *Mov. Disord.* **30**, 1591–1601 (2015). 26474316 10.1002/mds.26424

[30] Hasan, H., Athauda, D. S., Foltynie, T. & Noyce, A. J. Technologies assessing limb Bradykinesia in Parkinson’s disease. *J. Parkinsons Dis.* **7**, 65–77 (2017). 28222539 10.3233/JPD-160878 PMC5302048

[31] Espay, A. J. et al. Impairments of speed and amplitude of movement in Parkinson’s disease: A pilot study. *Mov. Disord.* **24**, 1001–1008 (2009). 19230031 10.1002/mds.22480

[32] Bologna, M., Paparella, G., Fasano, A., Hallett, M. & Berardelli, A. Evolving concepts on bradykinesia. *Brain* **143**, 727–750 (2020). 31834375 10.1093/brain/awz344 PMC8205506

[33] Denny-Brown D. Diseases of the basal ganglia. Their relation to disorders of movement. *The Lancet*. 276. 10.1016/S0140-6736(60)92353-9. (1960). 10.1016/s0140-6736(60)92353-9 13721915

[34] Bergman H. The hidden life of the basal ganglia: At the base of brain and mind. *The hidden life of the basal ganglia: At the base of brain and mind*. Published online 2021.

[35] Kupsch, A. et al. Pallidal deep-brain stimulation in primary generalized or segmental dystonia. *N. Engl. J. Med.* **355**, 1978–1990 (2006). 17093249 10.1056/NEJMoa063618

[36] Accolla, E. et al. Gender differences in patients with Parkinson’s disease treated with subthalamic deep brain stimulation. *Movement Disorders*. 22. 10.1002/mds.21520 (2007). 10.1002/mds.21520 17469208

[37] Golfrè Andreasi, N. et al. Short- and long-term motor outcome of STN-DBS in Parkinson’s Disease: focus on sex differences. *Neurol. Sci.* **43**, 1769–1781 (2022). 34499244 10.1007/s10072-021-05564-w

[38] Marceglia, S. et al. Gender-related differences in the human subthalamic area: A local field potential study. *Eur. J. Neurosci.* **24**, 3213–3222 (2006). 17156382 10.1111/j.1460-9568.2006.05208.x

[39] Wada, Y., Takizawa, Y., Zheng-Yan, J. & Yamaguchi, N. Gender differences in quantitative EEG at rest and during photic stimulation in normal young adults. *Clin. EEG Neurosci.* **25**, 81–85 (1994). 10.1177/155005949402500209 8194192

[40] Brenner, R. P., Ulrich, R. F. & Reynolds, C. F. EEG spectral findings in healthy, elderly men and women — sex differences. *Electroencephalogr. Clin. Neurophysiol.* **94**, 1–5 (1995). 7530634 10.1016/0013-4694(94)00234-c

[41] Brière, M. È, Forest, G., Chouinard, S. & Godbout, R. Evening and morning EEG differences between young men and women adults. *Brain Cogn.* **53**, 145–148 (2003). 14607135 10.1016/s0278-2626(03)00097-6

[42] Cahill, L. Why sex matters for neuroscience. *Nat. Rev. Neurosci.* **7**, 477–484 (2006). *7:6*. 2006. 16688123 10.1038/nrn1909

[43] Federman, D. D. The biology of human sex differences. *N. Engl. J. Med.* **354**, 1507–1514 (2006). 16598047 10.1056/NEJMra052529

[44] Celec, P., Ostatníková, D. & Hodosy, J. On the effects of testosterone on brain behavioral functions. *Front Neurosci.* **9**, 12 (2015). 25741229 10.3389/fnins.2015.00012 PMC4330791

[45] Isaias, I. U., Alterman, R. L. & Tagliati, M. Outcome predictors of pallidal stimulation in patients with primary dystonia: The role of disease duration. *Brain* **131**, 1895–1902 (2008). 18567622 10.1093/brain/awn120

[46] Mills, K. A., Starr, P. A. & Ostrem, J. L. Neuromodulation for dystonia. Target and patient selection. *Neurosurg. Clin. N. Am.* **25**, 59–75 (2014). 24262900 10.1016/j.nec.2013.08.014

[47] Vasques, X., Cif, L., Gonzalez, V., Nicholson, C. & Coubes, P. Factors predicting improvement in primary generalized dystonia treated by pallidal deep brain stimulation. *Mov. Disord.* **24**, 846–853 (2009). 19199337 10.1002/mds.22433

[48] Isaias, I. U. et al. Factors predicting protracted improvement after pallidal DBS for primary dystonia: The role of age and disease duration. *J. Neurol.* **258**, 1469–1476 (2011). 21365458 10.1007/s00415-011-5961-9

[49] Dorszewska, J. Cell biology of normal brain aging: synaptic plasticity-cell death. *Aging Clin. Exp. Res* **25**, 25–34 (2013). 23740630 10.1007/s40520-013-0004-2

[50] Mora, F., Segovia, G. & del Arco, A. Aging, plasticity and environmental enrichment: structural changes and neurotransmitter dynamics in several areas of the brain. *Brain Res. Rev.* **55**, 78–88 (2007). 17561265 10.1016/j.brainresrev.2007.03.011

[51] Coxon, J. P. et al. Reduced basal ganglia function when elderly switch between coordinated movement patterns. *Cereb. Cortex* **20**, 2368–2379 (2010). 20080932 10.1093/cercor/bhp306

[52] Cabello, C. R., Thune, J. J., Pakkenberg, H. & Pakkenberg, B. Ageing of substantia nigra in humans: </br>cell loss may be compensated by hypertrophy. *Neuropathol. Appl Neurobiol.* **28**, 283–291 (2002). 12175340 10.1046/j.1365-2990.2002.00393.x

[53] Rudow, G. et al. Morphometry of the human substantia Nigra in ageing and Parkinson’s disease. *Acta Neuropathol.* **115**, 461–470 (2008). 18297291 10.1007/s00401-008-0352-8 PMC2431149

[54] Giguère, N., Nanni, S. B. & Trudeau, L. E. On cell loss and selective vulnerability of neuronal populations in Parkinson’s disease. *Front Neurol.* **9**, 383041 (2018). 10.3389/fneur.2018.00455 PMC6018545 29971039

[55] Larsen, B. & Luna, B. Adolescence as a neurobiological critical period for the development of higher-order cognition. *Neurosci. Biobehav Rev.* **94**, 179–195 (2018). 30201220 10.1016/j.neubiorev.2018.09.005 PMC6526538

[56] Branch, S. Y., Sharma, R. & Beckstead, M. J. Aging decreases L-type calcium channel currents and pacemaker firing fidelity in substantia nigra dopamine neurons. *J. Neurosci.* **34**, 9310–9318 (2014). 25009264 10.1523/JNEUROSCI.4228-13.2014 PMC4087208

[57] Zauber, S. E., Watson, N., Comella, C. L., Bakay, R. A. E. & Metman, L. V. Stimulation-induced parkinsonism after posteroventral deep brain stimulation of the globus pallidus internus for craniocervical dystonia. *J Neurosurg.* **110**, 229–233 (2009). 10.3171/2008.6.17621 18976055

[58] Lofredi, R. et al. Beta bursts during continuous movements accompany the velocity decrement in Parkinson’s disease patients. *Neurobiol. Dis.* **127**, 462–471 (2019). 30898668 10.1016/j.nbd.2019.03.013 PMC6520224

[59] Moro, E. et al. Bilateral globus pallidus stimulation for Huntington’s disease. *Ann. Neurol.* **56**, 290–294 (2004). 15293283 10.1002/ana.20183

[60] Diederich, N. J., Kalteis, K., Stamenkovic, M., Pieri, V. & Alesch, F. Efficient internal pallidal stimulation in Gilles de la Tourette sydrome: A case report. *Mov. Disord.* **20**, 1496–1499 (2005). 16037913 10.1002/mds.20551

[61] Anderson, C. J., Anderson, D. N., Pulst, S. M., Butson, C. R. & Dorval, A. D. Neural selectivity, efficiency, and dose equivalence in deep brain stimulation through pulse width tuning and segmented electrodes. *Brain Stimul.* **13**, 1040–1050 (2020). 32278715 10.1016/j.brs.2020.03.017 PMC7308191

[62] Reich, M. M. et al. Short pulse width widens the therapeutic window of subthalamic neurostimulation. *Ann. Clin. Transl. Neurol.* **2**, 427–432 (2015). 25909087 10.1002/acn3.168 PMC4402087

[63] Moldovan, A. S. et al. Less is more – Pulse width dependent therapeutic window in deep brain stimulation for essential tremor. *Brain Stimul.* **11**, 1132–1139 (2018). 29735344 10.1016/j.brs.2018.04.019

[64] Kroneberg, D., Ewert, S., Meyer, A. C. & Kühn, A. A. Shorter pulse width reduces gait disturbances following deep brain stimulation for essential tremor. *J. Neurol. Neurosurg. Psychiatry* **90**, 1046–1050 (2019). 30765417 10.1136/jnnp-2018-319427 PMC6820151

[65] Niklas Petry-Schmelzer, J. et al. A randomized crossover trial of short versus conventional pulse width DBS in Parkinson’s Disease. *medRxiv*. Published online June 27, 2021.06.20.21258955. 10.1101/2021.06.20.21258955 (2021).

[66] Horn, A. et al. Optimal deep brain stimulation sites and networks for cervical vs. generalized dystonia. *Proc. Natl. Acad. Sci. USA*. 119. 10.1073/PNAS.2114985119 (2022). 10.1073/pnas.2114985119 PMC9168456 35357970

[67] Acevedo, G. et al. VisionMD: an open-source tool for video-based analysis of motor function in movement disorders. *NPJ Parkinsons Dis.* **11**, 27 (2025). 39900649 10.1038/s41531-025-00876-6 PMC11790922

[68] Pollo, C. et al. Magnetic resonance artifact induced by the electrode Activa 3389: An in vitro and in vivo study. *Acta Neurochir (Wien)*. 146. 10.1007/s00701-003-0181-4 (2004). 10.1007/s00701-003-0181-4 14963749

[69] Hemm, S. et al. Contact position analysis of deep brain stimulation electrodes on post-operative CT images. *Acta Neurochir. (Wien.)* **151**, 823–829 (2009). 19444372 10.1007/s00701-009-0393-3

[70] Yelnik, J., Bardinet, E., Dormont, D., Neuroimage, G. M. 2007 undefined. A three-dimensional, histological and deformable atlas of the human basal ganglia. I. Atlas construction based on immunohistochemical and MRI data. *ElsevierJ Yelnik, E Bardinet, D Dormont, G Malandain, S Ourselin, D Tandé, C Karachi, N AyacheNeuroimage, 2007•Elsevier*. 10.1016/j.neuroimage.2006.09.026 17110133

[71] Åström, M. et al. Relationship between neural activation and electric field distribution during deep brain stimulation. *IEEE Trans. Biomed. Eng.* **62**, 664–672 (2015). 25350910 10.1109/TBME.2014.2363494

[72] Reich, M. M. et al. A brain network for deep brain stimulation induced cognitive decline in Parkinson’s disease. *Brain* **145**, 1410–1421 (2022). 35037938 10.1093/brain/awac012 PMC9129093

[73] Keezer, M. R., Wolfson, C. & Postuma, R. B. Age, gender, comorbidity, and the MDS-UPDRS: Results from a population-based study. *Neuroepidemiology* **46**, 222–227 (2016). 26967747 10.1159/000444021

