# Supplementary material for: Bradykinesia induced by pallidal neurostimulation in dystonia: clinical risk factors and anatomical mapping
Source: NPJ Parkinsons Dis. 2025 Oct 23;11:308. doi: 10.1038/s41531-025-01177-8 (PMC12550046; doi:10.1038/s41531-025-01177-8)
Supplement: Supplementary file 1 — Supplementary Tables [file 41531_2025_1177_MOESM1_ESM.pdf]

Pat ID/Sex	*Dystonia Type*	*Age at Onset [yrs]*	*Age at Surgery [yrs]*	*Disease Duration [yrs]*	*Responder*	*Mean Amplitude [mA]*	*Mean Pulse Width [μs]*	*Mean Frequency [Hz]*
001 / m	CER	43	60	17	bad	2.6	260	130
002 / m	SEG	49	54	5	average	2.25	210	130
003 / m	CER	37	43	6	average	4.3	210	130
004 / m	CER	57	69	12	super	6	105	160
005 / m	GEN	9	21	12	average	4	105	155
006 / m	GEN	49	61	12	super	3,3	90	200
007 / m	GEN	33	61	28	average	4.7	105	180
008 / m	GEN	20	43	34	average	2.4	90	167
009 / m	GEN	56	66	10	super	3.4	90	130
010 / m	GEN	40	47	7	super	5.1	120	130
011 / m	CER	35	46	11	bad	4.2	120	180
012 / m	CER	17	31	14	non	3.7	90	130
013 / m	GEN	12	48	36	non	3.7	210	180
014 / m	GEN	16	38	22	super	3.3	105	180
015 / m	CER	56	67	11	super	2,4	120	180
016 / m	GEN	32	43	11	average	4	120	185
017 / m	GEN	10	42	32	super	3.4	90	150
018 / m	CER	32	41	9	bad	5	90	160
019 / m	GEN	11	61	50	non	2	60	130
020 / m	GEN	23	42	19	super	1.6	90	130
021 / m	CER	54	68	14	average	2.4	90	180
022 / m	CER	39	51	12	bad	2.7	90	130
023 / m	CER	60	62	2	average	3.3	120	130
024 / m	GEN	4	20	16	super	3.4	90	130
025 / m	CER	38	52	14	average	5.5	60	180
026 / m	GEN	7	34	27	average	3.9	120	130
027 / m	GEN	-	35	-	non	2.3	90	130
028 / m	CER	59	74	24	average	5.7	120	180
029 / f	CER	42	62	20	super	3.3	210	130
030 / f	GEN	65	70	5	non	3.5	150	130
031 / f	CER	44	48	4	average	3.1	90	180
032 / f	CER	60	70	10	average	4.6	120	130
033 / f	CER	30	41	11	average	2.6	90	180
034 / f	CER	49	54	5	super	3.8	60	130
035 / f	CER	49	50	1	super	3.4	90	130
036 / f	CER	-	70	-	average	3.1	105	180
037 / f	CER	56	66	10	non	4.3	105	180
038 / f	GEN	19	23	4	non	1.9	105	180
039 / f	GEN	7	26	19	super	4.4	90	130
040 / f	GEN	52	57	5	super	5.6	90	180
041 / f	CER	62	66	4	average	3.3	60	180
042 / f	CER	42	50	8	non	3.5	60	160
043 / f	GEN	52	67	15	average	2.3	195	150
044 / f	CER	31	63	33	bad	4.4	90	130
045 / f	CER	48	52	3	bad	3.5	105	130
046 / f	SEG	54	68	14	non	1.7	180	145
047 / f	CER	63	71	8	average	5.5	90	130
048 / f	SEG	53	62	9	average	2.3	120	180
049 / f	CER	46	57	11	non	1.1	60	180
050 / f	GEN	56	64	8	average	1.3	105	130
051 / f	GEN	6	27	21	average	3.9	120	185
052 / f	CER	37	45	8	average	3.9	105	180
053 / f	GEN	29	60	31	average	3.9	120	150
054 / f	GEN	5	48	43	average	3.5	90	130
055 / f	GEN	8	42	36	bad	3	105	130
Mean	**-**	**37**	**52**	**12**	-	**3.4**	**105**	**150**

Supplementary Table 1: This table presents a summary of the retrospective cohort studied in the project, including patient study ID, sex, dystonia type (CER for cervical, GEN for generalized, and SEG for segmental), age at surgery, age of onset, disease duration, responder status (bad, average, super, or non), and deep brain stimulation (DBS) parameters (amplitude, pulse width, and frequency). The last row displays the mean values for numerical variables, providing a rough description of the cohort.

Task	Kinematic Parameter	Spearman's Rho	P-value
Hand Opening	Movement Speed	−0.563	0.071
	Movement Frequency	−0.719	0.013
	Movement Amplitude	−0.233	0.490
	Amplitude Decay	−0.330	0.322
	Velocity Decay	−0.449	0.167
Finger Tapping	Movement Speed	−0.917	< 0.001
	Movement Frequency (Rate)	−0.369	0.265
	Movement Amplitude	−0.788	0.004
	Amplitude Decay	−0.147	0.665
	Velocity Decay	0.014	0.968

Supplementary Table 2: Correlation between Clinical Bradykinesia Scores and Kinematic Parameters in Cohort II
Correlations were assessed using Spearman's rank correlation (rho) between the clinical bradykinesia score (sum of MDS-UPDRS Part III items 3.4-3.8) and the mean kinematic parameters per patient, both measured under the full chronic stimulation condition (N=11). Significant p-values ($p < 0.05$) are highlighted in bold.
